# Blue–Green Infrastructure for Flood and Water Quality Management in Southeast Asia: Evidence and Knowledge Gaps

**DOI:** 10.1007/s00267-021-01467-w

**Published:** 2021-04-16

**Authors:** Perrine Hamel, Leanne Tan

**Affiliations:** Asian School of the Environment, Nanyang Technological University, Singapore, Singapore

**Keywords:** Stormwater management, Natural infrastructure, Nature-based solutions, Flood risk, Ecosystem-based adaptation

## Abstract

In Southeast Asia, projections of rapid urban growth coupled with high water-related risks call for large investments in infrastructure—including in blue–green infrastructure (BGI) such as forests, parks, or vegetated engineered systems. However, most of the knowledge on BGI is produced in the global North, overlooking the diversity of urban contexts globally. Here, we review the literature on BGI for flood risk mitigation and water quality improvement in Southeast Asian cities to understand the scope of practical knowledge and identify research needs. We searched for evidence of local types of BGI in peer-reviewed and grey literature and assessed the performance of BGI based on hydrological, societal, and environmental metrics. The body of literature on BGI in Southeast Asia is small and dominated by wealthier countries but we found evidence of uptake among researchers and practitioners in most countries. Bioretention systems, constructed wetlands, and green cover received the most attention in research. Evidence from modelling and laboratory studies confirmed the potential for BGI to address flooding and water quality issues in the region. However, practical knowledge to mainstream the implementation of BGI remains limited, with insufficient primary hydrological data and information on societal and environmental impacts. In addition, the performance of BGI in combination with grey infrastructure, under climate change, or in informal settlements is poorly studied. Future research and practice should focus on producing and sharing empirical data, ultimately increasing the regional knowledge base to promote efficient BGI strategies.

## Introduction

Whether it is mitigating flood risk, improving access to clean water or treating urban water effluents, managing water is a top priority for all cities in the world. Nature-based solutions are gaining traction among local governments, multilateral and non-governmental organizations to deliver efficient and sustainable urban water management (Asian Development Bank 2019; Brears 2018; Liu and Jensen 2018; World Wildlife Fund 2016). These solutions leverage ecosystem services, the benefits provided by nature, to improve water management by restoring a more natural water cycle, for example increasing infiltration, evapotranspiration and pollutant removal (Eckart et al. 2017; Fletcher et al. 2013; Liao et al. 2017). Importantly, nature-based solutions provide a range of additional services such as reducing urban heat island, sequestering carbon and providing aesthetic or recreational value to a city (Depietri and McPhearson 2017; Keeler et al. 2019; Lourdes et al. 2021; Venkataramanan et al. 2019). The multifunctionality of nature-based solutions aligns well with a systemic or ‘integrated’ approach to water management, one that integrates multiple stakeholders and multiple solutions to increase resilience to water-related hazards (Fletcher et al. 2015; Mitchell 2006; Oral et al. 2020).

Nature-based solutions rely on blue and green infrastructure (BGI), or ‘natural infrastructure’, the ‘interconnected network of natural and semi-natural elements capable of providing multiple functions and ecosystem services’, ranging from green spaces to riparian and coastal vegetation, street trees and engineered systems such as bioretention or green roofs (Bartesaghi Koc et al. 2017; Benedict and McMahon 2006). The multiple services derived from BGI in a given city depend on a range of social, ecological and technological factors (Keeler et al. 2019). For example, climate and soil characteristics determine how much water is stored in a watershed, making forests more or less effective at storing and slowly releasing water in the dry season (Brauman 2015; Browder et al. 2019). Socio-technical decisions about the water supply system—e.g. reliance on surface versus groundwater—also moderate the value of BGI, by changing the demand of the local groundwater recharge.

Despite the importance of understanding local factors, most of the academic knowledge on BGI was generated in the global North, leaving knowledge gaps about the performance of green infrastructure in other regions (Keeler et al. 2019; Nagendra et al. 2018; Song et al. 2017). Filling these gaps is particularly important in Southeast Asia, where projections of rapid urban growth and important risks of flooding and water pollution make integrated urban water management critical. Southeast Asian countries currently show a ‘low’ to ‘medium low’ level of integration, except the Philippines (‘medium high’) and Singapore (‘very high’), highlighting the potential for BGI to be further incorporated in urban water management (UNEP-DHI Centre on Water and Environment 2020).

It is estimated that over 500bn USD are needed to improve water infrastructure in Southeast Asia, with country-level projections varying from 4.6 in Cambodia to 209bn in Indonesia (data excluding Timor Leste, Lao PDR and Brunei) (Global Infrastructure Hub 2020). The share of BGI in these investments will depend on the capacity of public and private sectors to consider and implement nature-based solutions, motivated by regulatory changes (e.g. water quality standards), water security, increased flood risk or ecological considerations (Liao et al. 2017). In practice, it will also require addressing institutional, financial and knowledge barriers to BGI implementation (Sarabi et al. 2020, 2019; Wamsler et al. 2020). The motivation for this paper is to review technical knowledge gaps for two categories of services in Southeast Asia: flood risk mitigation and water quality improvement, focusing on hydrological and engineering knowledge rather than governance or urban planning processes. For eleven Southeast Asian countries, we reviewed the peer-reviewed and grey literature on BGI and extracted information on hydrologic performance (whether BGI is effective at providing water-related services) and practical considerations to design and implement BGI in the region. We asked:

- what type of BGI for flood risk and water quality improvement is studied in Southeast Asian cities, and in which countries?
- what is the evidence of hydrologic performance for such BGI?
- what information is available to design and implement such BGI in practice (considering the ecological and socio-technological context of Southeast Asia)?

In the next sections, we describe the characteristics of Southeast Asian countries (‘Southeast Asian Ecological and Socio-technical Context’) and our methodological framework to review the regional literature (‘Methods’). We then organize our findings and discussion according to the three questions above, namely geographic distribution and types of BGI found in the literature, hydrologic performance of BGI and design and implementation factors. By contrasting our findings with the global literature, we conclude with a summary of research gaps and future research directions in the region (‘Key Knowledge Gaps’).

## Southeast Asian Ecological and Socio-technical Context

Several ecological and environmental features distinguish Southeast Asia from most global North countries. First, the climate—tropical rainforest or tropical monsoon (Beck et al. 2018)—is hot and humid, with high annual precipitation and frequent intense events. This has implications for BGI as more water storage capacity is needed to manage water (Eckart et al. 2017), whether it is for flood control or water quality management. For example, the intensity of a 1-h storm occurring every 2 years in Singapore is equivalent to a storm occurring every 100 years in New York (Cornell University 2015; Public Utilities Board Singapore 2013). It makes the area particularly prone to flood hazards, with Thailand, Cambodia, Vietnam and the Philippines being among the most flood-impacted populations around the world (Hu et al. 2018). Climate change will also exacerbate these trends (e.g. Kefi et al. 2018; Wang et al. 2017). Second, Southeast Asia comprises tropical and subtropical rainforest, dry forest and monsoon forest, species that are understudied in the global literature (Song et al. 2017). Most Southeast Asian trees are evergreen, with only a few deciduous species found in the dry forests (e.g. in Myanmar), resulting in little seasonal variation in ecological functions other than due to climate. Third, Southeast Asian soils generally have high clay contents and medium to low permeability (Acrisol–Alisol types, Chappell et al. 2007). Although this suggests that forests may be less effective at retaining water, the large swaths of forests with highly porous topsoil still provide high drainage rates—making them important to protect for water security (Estoque et al. 2019). A final important characteristic of the Southeast Asian landscapes is the presence of large river deltas, including the Mekong, the Irrawaddy and the Chao Phraya deltas, which all comprise large wetland areas. The flat topography of these regions exposes urban and rural settlements to frequent flooding (e.g. Siripong et al. 2000).

From a socio-technological standpoint, Southeast Asian cities range broadly in population density, gross domestic product per capita and governance efficiency (Table S1, Supplementary Information and Lourdes et al. 2021). These characteristics influence the range of BGI that are possible to implement, based on cost and space requirements, among others. Weak urban governance means that official plans are rarely implemented, with market forces rather driving what is built on the ground (Yap 2018). The resulting ‘tetris-like’ urban sprawl, described by some scholars in China and Southeast Asia (Hedglin 2015; Yap 2018), is at odds with integrated urban water management, which requires finding synergies and complementarities in various components of the water system (Liu and Jensen 2018). In fact, few Southeast Asian cities have an integrated urban water management plan and sanitation remains very low in most countries (Rahmasary et al. 2019). Apart from Singapore and Malaysia, Southeast Asian countries have a low sewerage cover with on average 17.3% of urban dwellers being connected to a sanitation sewer network (World Health Organisation and UNICEF 2017, see Table S1 for national statistics). Cities rarely have separate stormwater and sanitary sewer systems, with the notable exception of Singapore and, to a lesser extent, cities in Malaysia, Brunei and Vietnam. Most cities include open canals that drain both stormwater and wastewater, often to the nearest river—making water purification an essential service to remove pollution. Subsidence due to groundwater pumping exacerbates flooding issues, as it has been demonstrated in Jakarta, Bangkok or Ho Chi Minh City (Erkens et al. 2015). BGI that increases infiltration may mitigate this issue, while also replenishing groundwater resources for consumption.

Finally, Southeast Asian cities are characterized by high levels of informality. Informal settlements are defined as urban areas developed outside the legal systems and lacking ‘risk-reducing infrastructure (paved roads, storm and surface drainage, piped water, etc.) and services relevant to resilience (including healthcare, emergency services and rules of law)’ (Satterthwaite et al. 2020). More than 370 million people live in informal settlements in Southeastern and Eastern Asian cities, making up to 50% of the urban population in some countries (Table S1). These settlements are particularly vulnerable to hydrologic hazards due to the lack of infrastructure and proximity to water bodies. In many cities, informal dwellers both impact and rely on services related to rivers (Vollmer and Grêt-Regamey 2013), making them important actors of river management. The uncontrolled growth of informal settlements may exacerbate water management issues by reducing ecosystem services such as water purification and water retention (Harriden 2012). Inexistent or limited waste collection and sanitation services also severely impacts water quality.

## Methods

### Review Framework and Search Terms

**Fig. 1:**
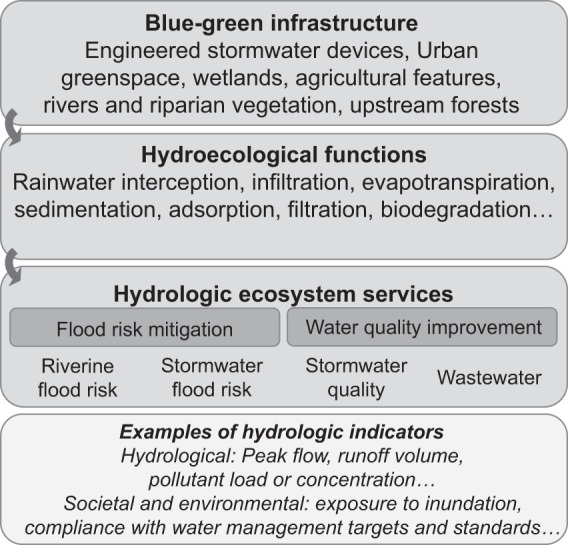
Framework to study the performance of blue–green infrastructure on two hydrological ecosystem services in this study. Blue–green infrastructure performs several hydroecological functions that produce four key services. Additional services outside the scope of our review include coastal flood risk (by marine and coastal blue–green infrastructure) and water supply

Given our focus on hydrological performance and technical design, we use the term BGI throughout the review, which highlights the *structural* elements of nature-based solutions and the broad focus on urban water management rather than stormwater specifically (cf. discussion of alternative terms by Fletcher et al. 2015; Moosavi et al. 2021). BGI performs a range of ecohydrological functions (infiltration, sedimentation, biodegradation), which provide services (Brauman 2015; Tallis et al. 2012). Using Brauman’s framework (2015), we focused on risk mitigation and supporting services. Two are related to flood risk (riverine and stormwater flood mitigation) and rely on the capacity of vegetated systems to intercept, infiltrate and retain rainwater during storms, which reduces runoff volume and delays peak flows. The other two services relate to water quality (stormwater and wastewater management), relying on interception, infiltration and retention, as well as pollutant reduction through sedimentation in water bodies, filtration and biodegradation (Fig. 1).

We searched all scientific databases on the Web of Knowledge platform for peer-reviewed articles published before June 2020 on urban water management in Southeast Asian countries. Our search terms reflected our interest in a broad range of BGI—including site-scale to watershed-scale ecosystems—although we excluded marine ecosystems for coastal flood risk mitigation. We included the name of Southeast Asian countries and river basins and the generic terms:

‘Blue green infrastructure’ OR ‘green infrastructure’ OR ‘natural infrastructure’ OR ‘nature-based solutions’ OR ‘river restoration’ OR ‘river rehabilitation’

together with specific types of BGI (e.g. ‘raingarden’, ‘green roof’, ‘paddies’, ‘wetlands’), processes (‘bioretention’, ‘biofiltration’) and fields of research and practice (‘watershed management’, ‘flood risk’, ‘water sensitive urban design’, ‘low impact development’, Supplementary Information).

We selected studies that (i) addressed at least one of the four services listed above, (ii) directly affected urban areas in Southeast Asia and (iii) provided evidence of the hydrologic performance of BGI or technical considerations for design and implementation. We also reviewed the grey literature, including reports and guidelines from websites of major conservation or development organisations, think tanks or multilateral banks, extracting information on implementation guidelines or hydrologic performance from regional case studies. Additional details on our search criteria and methods can be found in Supplementary Information.

### Types of BGI

We classified the main types of BGI in four main categories used in previous research (Keeler et al. 2019; Liao et al. 2017): urban green space, engineered stormwater devices (including bioretention systems, bioswales, green roofs, retention and detention ponds), wetlands (constructed and natural) and watershed management features (forests, rivers and riparian vegetation and agricultural features) (Table 1). This categorization reflects the range of measures from watershed-scale (forests) to site-scale (engineered stormwater devices). In reviewing the literature, we paid specific attention to local types of BGI, including traditional architectural features. Such BGI specific to the region are of particular importance since they are likely to be more adequate and accepted in an inclusive approach to nature-based solutions (Frantzeskaki 2019).

**Table 1:** Types of blue–green infrastructure (BGI) studied in Southeast Asia and examples of regional implementations

	Description	Regional examples
Green cover in urban areas (15%)	**Green cover** or green space includes urban parks, gardens, green belts or corridors (they also overlap with green roofs, described below). Increasing green spaces in urban areas helps reduce peak flows and reduce stormwater pollution by reducing runoff volume, while also providing opportunities for recreation.	• Malaysia: ‘pocket parks’, of modest dimensions, provide many benefits with low space requirements (Balai Kerishnan et al. 2020). • Indonesia: *telajakan* are ‘strips of traditional green space between the wall of a housing compound and a ditch/pedestrian path in a roadside’ (Kato et al. 2017). Despite their aesthetic and cultural value, their presence is declining.
Engineered stormwater devices (49%)	**Bioretention systems, bioswales** (34%)	
	Bioretention systems, or rain gardens, are engineered devices collecting stormwater to reduce runoff and treat pollutants. They comprise several soil and vegetation layers, designed to treat, store and/or infiltrate stormwater (e.g. rain gardens, biopores) and/or convey it (bioswales).	• Bioretention systems are increasingly used in Southeast Asian cities (Hermawan et al. 2020; Sidek et al. 2013; Wang et al. 2019). • Indonesia: Biopores, tubular holes in the soil, are an example of infiltration systems (Drosou et al. 2019; Setiawan and Rohmat 2018).
	**Green roofs** (12%)	
	Green roofs are vegetated systems collecting rain that falls on buildings. They have layers similar to bioretention systems, including an impervious membrane. Green roofs help reduce stormwater volume and pollution but have a limited effect on peak flows.	• Many implementation in particular in Thailand, Malaysia, Singapore and Indonesia (Lim and Lu 2016; Maryati and Humaira 2017). • Thailand: the Centenary Park at Chulalongkorn University is a prominent example of an urban park used as NBS to retain flood waters (Holmes 2019).
	**Retention ponds or sedimentation basins** (6%)	
	Sedimentation basins or ponds filter and capture coarse sediments and litter, mainly from storm events or sewerage. Sedimentation basins can also be used as a pre-treatment method for wastewater to remove larger suspended solids before being fed into wetlands. Retention ponds are artificial water bodies that store excess runoff during a storm.	• Thailand: flood control and irrigation using retention ponds or ‘monkey cheeks’, known in Thai as Kaem Ling (King Rama IX was inspired by how monkeys store bananas in their pouch for later consumption). Water is stored in a network of retention ponds and irrigation canals (Ditthabumrung and Weesakul 2019; World Wildlife Fund 2016) to be used in the dry season.
Wetlands (41%)	**Wetlands** are ecosystems flooded with water such as mangroves, peatlands and man-made ecosystems featuring shallow water bodies and vegetation (‘constructed wetlands’). They help reduce peak flows, store stormwater and remove nutrients and pollutants from wastewater. Wetlands are also useful in mitigating coastal flooding, which may reduce urban flooding.	• Cambodia: Phnom Penh partly relies on natural wetlands to treat municipal wastewater (Irvine et al. 2015). • Constructed wetlands are gaining popularity in many places, in particular constrained island environments (Brix et al. 2011; Danley-Thomson et al. 2016; Shutes 2001). • Peatlands also form an important part of the landscape and are thought to help store flood waters (Klepper 1992; Sumarga et al. 2016).
Watershed-scale features (17%)	**Agricultural landscape features** (6%)	
	Rice paddy fields are common in Southeast Asia, and fields must be flooded, which means they may increase floodwater storage. Rice paddies also have the potential to treat domestic wastewater. Canals for floodwater diversion or irrigation offer an alternative channel for excess water to be stored during a potential flood event (Huu Loc et al. 2020).	• Rice paddies are common in Southeast Asia, particularly in the Mekong river basin (Masumoto et al. 2008; Rambonilaza and Neang 2019). • Features for floodwater diversion are common in Southeast Asia, including urban water bodies (Maryati and Humaira 2017; Wolf et al. 2020) and furrows (Watkin et al. 2019).
	**Upstream forests, rivers, riparian vegetation** (12%)	
	Forests upstream of urban areas reduce runoff volume and can help mitigate riverine flooding. River restoration, or river reclamation, also plays an important role both hydraulically—increasing room for water and reducing flow velocity—and socially—reducing exposure to flood hazard.	• Tropical forests occupy a large part of Southeast Asia although they are severely degrading (Hughes 2017). • Singapore: the Kallang River restoration project has been praised for its innovative use of green spaces in urban areas (Dreiseitl et al. 2015). Other restoration projects are under construction, for example in Indonesia (Lin et al. 2016).

Percentages in parentheses indicate the number of papers focusing on the specific BGI (out of 109 papers). They do not add up to 100% as some papers had several types of BGI

### Hydrologic Performance

For peer-reviewed literature, we extracted information on hydrologic performance, including commonly used indicators of peak flow, runoff volume reduction, pollutant removal efficiencies (total nitrogen, phosphorus and suspended solids—TN, TP, TSS—as well as biological oxygen demand, BOD, for wastewater). We distinguished between empirical data (including models with validation) and modelling results without validation, which typically have higher uncertainty, and systematically reported the former types of studies (Tables S3–S5). We also reported information on societal and environmental benefits (flood risk reduction, ecological improvement due to water quality improvements, Fletcher et al. 2013; Liao et al. 2017). Stormwater management best practices focus on mimicking the pre-development water cycle—reducing runoff volumes and increasing infiltration—to improve ecological impacts (Fletcher et al. 2013; Shuster et al. 2005). We therefore used the water budget at a watershed scale as a possible metric for ecological improvement. Additional metrics include compliance with standards (e.g. from the International Organization for Standardization) or guidelines (e.g. urban water management guidelines developed by government agencies). We also identified specific factors (e.g. vegetation type, climate) reported in the studies that may explain performance variability. Finally, we identified the studies that comprised hydrologic measurements as opposed to modelling studies with limited validation.

### BGI Design and Implementation Factors

Barriers to BGI implementation include limited knowledge on implementation or effectiveness, limited physical space, weak and siloed governance, ‘path dependency’ or resistance to change and inadequate financial resources (Sarabi et al. 2020, 2019; Wamsler et al. 2020). Given our focus on hydrological performance, we focused on the first category—knowledge for implementation and effectiveness—and extracted information on four design factors of particular importance: consideration of grey infrastructure (‘hybrid’ infrastructure), climate change, co-benefits (i.e. benefits from BGI that are not related to their hydrological functions) and implementation in informal settlements.

## Results

We compiled a list of 109 peer-reviewed papers (full list in Supplementary Information). Of the peer-reviewed papers, 51 addressed stormwater management, including 39 on stormwater quantity and 20 on quality, 42 papers addressed wastewater management, and 20 addressed riverine flooding. In addition to the scientific literature, we listed 27 guidance documents in the ‘grey’ literature that represent an important source of information on BGI potential and implementation. For example, the Asian Development Bank’s (2016) case studies on Nature-Based Solutions for Building Resilience in Towns and Cities detail the applicability of different types of BGI in the Greater Mekong subregion, including specific benefits and caution notes. Singapore’s ABC Waters Programme and the Australia-Indonesia Centre produced useful guidelines and detailed case studies (Payne et al. 2019; Public Utilities Board Singapore 2018). Technical and strategy reports also provide useful local lessons on BGI adoption for consideration in other Southeast Asian countries. The full list in Supplementary information also comprises international guidelines applicable to the region (Browder et al. 2019; Colgan et al. 2017; World Bank 2016; World Wildlife Fund 2016).

### Types of BGI and Geographic Distribution of the Peer-reviewed Literature

We found references to the four main categories of BGI described in Table 1 in the peer-reviewed literature. Engineered stormwater systems were the most-studied BGI (*n* = 53), closely followed by wetlands (*n* = 45, including a majority of constructed wetlands), watershed management features (*n* = 19) and urban green cover (*n* = 16). Examples of regional implementations suggest that some types of BGI are specific to the region and therefore rarely found in the global North literature. These include some agricultural features (rice paddies), or architectural idiosyncrasies (*telajakans* in Bali, Indonesia, Table 1).

**Fig. 2:**
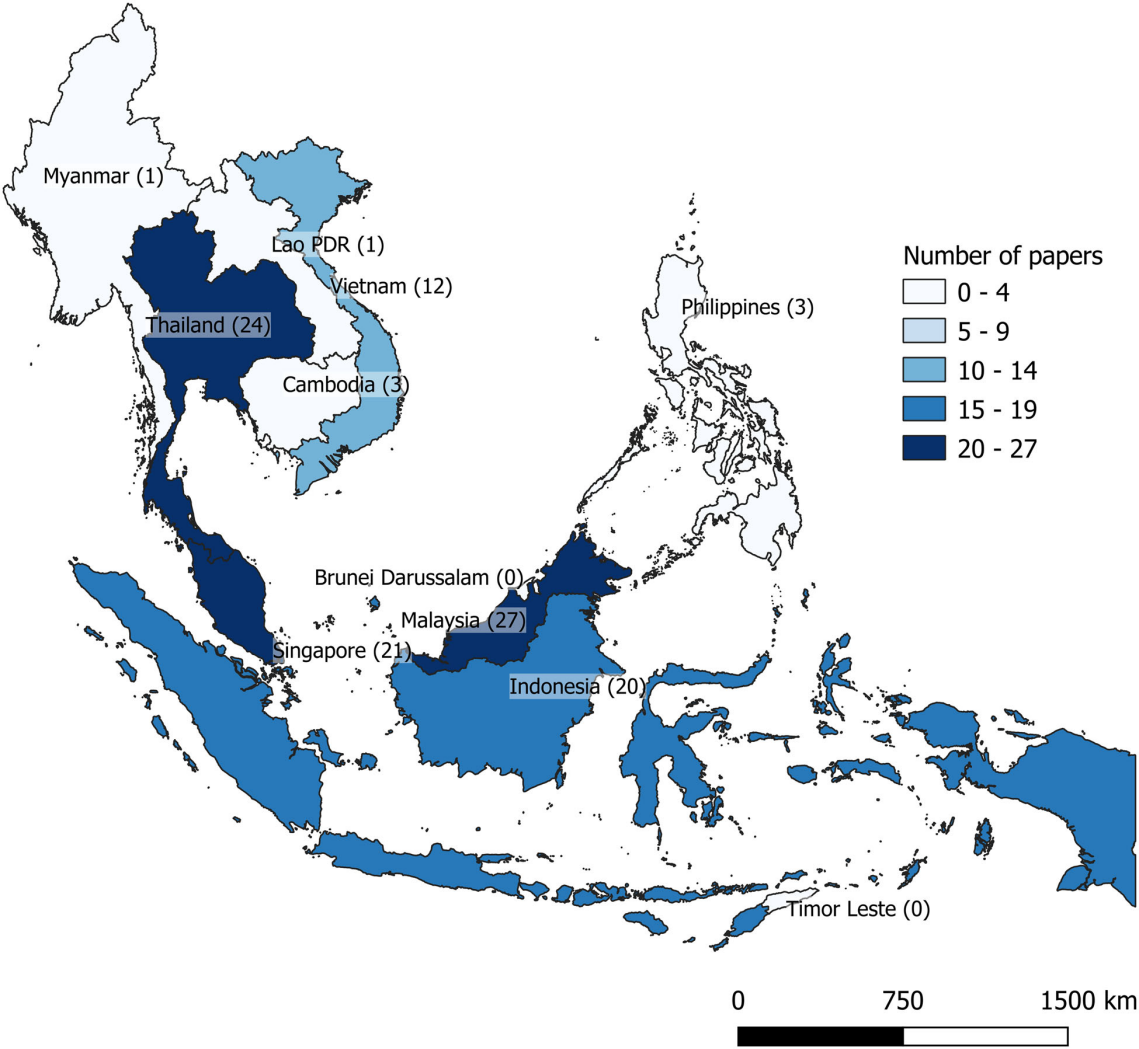
Map of Southeast Asia colour-coded for number of publications found in our systematic search

High- and upper-middle-income countries generally have the highest number of peer-reviewed publications (Fig. 2), and no publications were found for Timor Leste and Lao PDR. We found the highest number of publications related to flood management in Indonesia and Singapore (*n* = 25). Nearly half (*n* = 17) of the peer-reviewed publications on wastewater management were from Thailand, with most of them citing the need for wastewater management in cities affected by tourism.

### Hydrologic Performance

#### Riverine Flood Risk Reduction

The most-studied type of BGI for riverine flooding was upstream forests. Research in the Ciliwung River, Indonesia and Kelantan River, Malaysia confirmed the effect of forests on peak flow attenuation—although the effect remained limited for large precipitation events (Abdulkareem et al. 2018; Asdak et al. 2018). In a modelling study, Asdak et al. (2018) found that the reduction of forested area from 58 to 34% in the Citarum watershed resulted in the increase of peak flow by around 14%. Abdulkareem et al. (2018) investigated four river basins in Malaysia and found that (observed) peak flow increased by 8–39% from 1984 to 2002 due to forest cover reducing by 19–59%. In Thailand, Sriwongsitanon and Taesombat (2011) showed that forests reduce peak flow and runoff coefficients for small events but they can increase runoff coefficients—the ratio between runoff and precipitation—for large events. The authors explain this effect by the higher soil moisture retention in forests, increasing total runoff from large events occurring after a wet period. In a modelling study in the Tamontaka river basin, Philippines, Buisan et al. (2019) reported that forest conversion to agriculture by 22% would increase peak flow by about 10%. None of the reviewed articles provided empirical evidence of the performance of forest restoration projects and many quantitative studies relied on uncalibrated model simulations (five of eight studies for upstream forests).

Despite the high potential of river restoration projects (Ozment et al. 2019), we found little evidence of such projects in Southeast Asia. In a modelling study, Lin et al. (2016) found that revegetation and implementation of retention basins along the Ciliwung River in Jakarta, Indonesia, would have a negligible effect on peak flow for the 2-year return flood, due to inability to store large volumes of water. Although implementation of BGI was less effective than grey infrastructure (canalization) from a hydraulic standpoint, it still reduced inundation extent in several settlements along the river. Recent projects such as the river corridor improvement project in the Klang River tributaries, Malaysia (cf. project reports in Supplementary Information), or the Kallang River project, in Singapore, could add to the evidence base although we found little monitoring data in the literature, as noted by others (Lim and Lu 2016).

A few studies quantified the role of paddies and irrigation systems in flood risk mitigation. Masumoto et al. (2008) established that paddies around the Tonle Sap lake could store up to 17% of the flood volume, and irrigation systems up to 42% in the lower lands, for a large flood event. Ditthabumrung and Weesakul (2019) found that irrigation systems, including in ‘monkey cheeks’ in Northern Thailand could effectively reduce flooding by creating more flood storage. Agricultural land areas in the watersheds upstream of Bangkok, in the Chao Phraya basin, seem to play an important role in delaying and reducing peak flow (Siripong et al. 2000). Although we did not find quantitative data, some researchers also highlight the role of peatlands, a type of wetland common in Southeast Asia, in water storage. Drainage of peatlands for cultivation may lead to subsidence and increase flooding both in the plantation and potentially downstream (Klepper 1992; Sumarga et al. 2016).

#### Stormwater Flood Risk Reduction

Studies examining stormwater flooding focused on engineered systems, mostly bioretention systems and retention ponds but also green roofs. Payne et al. (2019) compiled the evidence and guidelines for the implementation of engineered systems in Bogor, Indonesia, which are relevant to most Southeast Asian cities. The authors highlight that empirical evidence in the region is scarce—only a few studies are cited to quantify the hydrological performance of stormwater systems—but they compile a useful list of existing resources to design engineered systems in the region (see Supplementary Information).

In Singapore, a review conducted by Lim and Lu (2016) concluded that systems in the Active Beautiful Clean (ABC) Waters programme, an initiative promoting BGI for water management in the country, provide average runoff retention performance for engineered devices. The authors conclude that there limited empirical evidence of the hydrologic performance of new BGI features implemented in the country. They also provide technical recommendations, e.g. that bioretention systems and wetlands should be designed to mitigate at least low magnitude flood risk (1-year flood event). Additional evidence from recent studies suggests that a bioretention system designed according to the ABC Waters programme guidelines could reduce peak flow by an average of 94% (Wang et al. 2017). Trinh and Chui (2013) found that a combination of green roof and bioretention systems can return peak runoff to pre-urbanisation levels, while Wang et al. (2018) found that up to 75% could be retained in bioretention cells. Peak flow was also reduced by 63% in a study by Goh et al. (2017); and by 33% in a study by Yau et al. (2017) (10-year average recurrence interval). Outside Singapore, we found other studies confirming the retention performance of BGI in a tropical climate, and sometimes in low permeability soils, with examples in Malaysia (Lai and Mah (2012); Rezaei et al. (2019)); Indonesia (Setiawan and Rohmat (2018)); Vietnam (Loc et al. 2015) and Thailand (Chaosakul et al. 2013; Majidi et al. 2019). Overall, we found empirical evidence of runoff volume retention by engineered systems ranging from 1 to 100%, with the low values corresponding to high intensity events (Table S3).

Among the factors explaining hydrological performance, rainfall intensity was examined in a few studies, with higher intensity leading to lower BGI efficiency (e.g. Azkarini et al. 2019; Majidi et al. 2019; Yau et al. 2017). However, evidence confirms that with appropriate design, BGI can reach high retention performance as illustrated in the studies cited above. To address local climate conditions, Yau et al. (2017) propose a revision of the design guidelines to consider runoff volume instead of the more traditional average recurrence interval (e.g. 3-month return), since the latter does not adequately capture the wide range in rainfall intensity in the Tropics.

We found little information on the type of vegetation that might increase retention and most studies use default parameters from non-tropical countries (e.g. for engineered systems in Majidi et al. (2019) or land use in Goh et al. (2017)). There was also limited information on long-term maintenance. Ong et al. (2012) found that some plant species may require manual irrigation, contrasting with water quality guidelines on plant selection (see next section). We did not find any information on the interactions between stormwater and riverine or coastal flooding.

#### Stormwater Quality Improvement

Engineered systems are commonly used to manage stormwater quality, in particular bioretention cells, with 14 out of the 21 reviewed studies on stormwater quality including this type of BGI. In their review focused on Singapore, Lim and Lu (2016) found low nutrient removal and leaching from rain gardens and constructed wetlands, but a good performance for TSS removal. Ong et al. (2012) and Wang et al. (2017) also found that TN and TP removal rates were lower in Singapore, due to insufficient storage capacity, leading to overflow, and incomplete denitrification. Compared to other countries, stormwater runoff in Singapore seems relatively low in nutrient content. Sim et al. (2008) found a good nutrient removal performance, up to 82% for TN and 83% for TP, of the Putrajaya constructed wetland, confirmed by laboratory studies. They note the lower performance during periods of heavy rains and the role of evapotranspiration in increasing nutrient concentration, which should inform the design of future wetlands. Green roofs also receive increasing attention for their role in reducing runoff, hence reducing stormwater quality issues (Chai et al. 2017; Kok et al. 2013), but also potential disservice (increasing nutrient loads, Vijayaraghavan et al. 2012). One study assessed the performance of a floating wetland (TN removal rates ranged from 7 to 67%), suggesting that this type of BGI has some potential to improve water quality in reservoirs (Chua et al. 2012). Despite an emerging body of literature examining optimal design and maintenance options of these systems, studies often call for more research on the performance of floating wetlands systems or innovative combinations of engineered systems in the Tropics (Lim and Lu 2016). Overall, we found empirical data for TSS removal ranging from 53 to 92%, TN removal from 25 to 82% and TP removal from 21 to 83% (Table S4).

Plant selection plays an important role in removal rates of engineered systems, and Hermawan et al. (2020) found that three native plant species could survive well and reach high treatment removal performance. A number of laboratory studies are available to guide plant selections, including a comprehensive study in Singapore (Loh 2012; Loh and Hunt 2013), and Australian studies reviewed by Payne et al. (2019).

#### Wastewater Quality Improvement

A large majority (33 of 40) of the reviewed papers on wastewater treatment use small-scale prototypes to determine the suitability of various plants for pollutant removal in constructed wetlands. For example, constructed wetlands planted with canna and heliconia were shown to have similar removal efficiencies, although canna grew better in domestic wastewater (Qomariyah et al. 2018). Other examples include the use of cocopeat (Danley-Thomson et al. 2016), reed and vetiver grasses (Nguyen et al. 2020) or rice (Kantawanichkul et al. 2003). Mangrove plantations were also investigated by Boonsong et al. (2003), who found that plantations provided similar treatment to existing mangrove forests (45–54% and 23–65% removal, respectively, for TN and TP). Several studies also examined other design options, e.g. roof wetland (Bui et al. 2014), or different feeding strategies (Ni et al. 2013).

Optimal design options for different types of effluents have been tested, such as domestic (Engida et al. 2020; Salih et al. 2017; Koottatep et al. 2001; Liamlaem et al. 2019), municipal landfill leachate in Thailand and Malaysia (Akinbile et al. 2012; Ogata et al. 2015; Sawaittayothin and Polprasert 2007), industrial effluent from the batik industry in Indonesia (Effendi et al. 2018; Rahmadyanti et al. 2020), seafood industry in Thailand (Yirong and Puetpaiboon 2004) or palm oil industry in Malaysia (Sa’At et al. 2019; Ujang et al. 2018), biochemical effluent (Meutia 2001; Vo et al. 2019) or agricultural wastewater in Malaysia and Thailand (Kantawanichkul et al. 2003; Liang et al. 2017; Pongthornpruek 2017). Studies in Singapore and Thailand also examined the role of constructed wetlands for pharmaceutical removal (Vo et al. 2019; Zhang et al. 2015, 2012). Only three studies examined the role of wetlands for greywater treatment: a horizontal flow wetland in treating greywater in Indonesia (Qomariyah et al. 2018), and two case studies in Thailand (Brix et al. 2007; Liamlaem et al. 2019), which all confirmed that wetlands could effectively treat water for non-potable reuse (Payne et al. 2019).

Only a limited number of papers studied full-scale systems, mainly in Thailand (Brix et al. 2011, 2007; Møller et al. 2012), Malaysia (Shutes 2001), Vietnam (Trinh et al. 2013) and Cambodia (Irvine et al. (2015). Among those, two constructed wetlands in Thailand provided nitrogen removal rates of 38 and 86%, and biological demand reduction by 72 and 87% (Brix et al. 2011; Møller et al. 2012). This may reflect the low number of constructed wetlands for wastewater management, as the grey literature also has few examples (the majority of wetlands in the grey literature were studied for coastal flood protection).

#### Societal and Environmental Benefits of BGI

Most of the reviewed papers assessed the biophysical functions of BGI, i.e. peak flow reduction, water retention and pollutant removal efficiency. Only a few studies examined the societal and environmental benefits—i.e. the actual (hydrologic) services (Fig. 1, bottom row). Few studies reported the effect of BGI on the water cycle, in part because many of them were conducted at the site scale. With regard to flood risk mitigation, studies rarely looked at the effect of BGI on inundation. Some exceptions include the study by Kefi et al. (2018), who found that the implementation of bioretention systems could reduce inundated areas by 59% and losses by 29% in an urban watershed in Vietnam. Analyzing 16 years of flood data in 31 basins in Malaysia, Tan-Soo et al. (2016) found robust evidence on the link between forest conversion to oil palm and rubber plantation and occurrence of flooding. Using an econometric approach, they estimate the effect of inland forest conversion to oil palm on number of days flooded, potential additional deaths and evacuations. Majidi et al. (2019) presented a map of inundation in a neighbourhood of Bangkok, Thailand although they expressed their modelling results as runoff volume and peak flow reduction, not flood impact. Pre-urbanisation peak flow may be used as a reference to show that the increase in flood hazard due to urbanisation has been mitigated, as demonstrated by Trinh and Chui (2013) in their study of a mix of green roof and bioretention systems. For water quality, certifications specifying acceptable effluents levels can be used a benchmark for BGI implementation. They were used in one study of domestic wastewater treatment by constructed wetlands in Sakon Nakhon, Thailand, showing that the wetlands were maintained and effective after 13 years of implementation and obtained the ISO9001 certification (Liang et al. 2017). Studies in Singapore also regularly refer to the ABC Waters guidelines.

Only a few studies report the economic value of BGI to inform the economic and financial feasibility of future projects. Agus et al. (2006) through a replacement cost approach, valued the retention service of paddies in the Citarum watershed, Indonesia, at USD 92.67 million per year or 51% of the total price of rice produced in the field. Ro et al. (2020) valued the water treatment service of a wetland in Phnom Penh, Cambodia, at about USD 3 million per year. Goh et al. (2017) found that every SGD10,000 of BGI features incorporated can reduce the runoff coefficients (e.g. by from 0.25% in green roofs to 3.5% in porous pavements).

### Design and Implementation Factors for BGI

#### Combining Grey Infrastructure and BGI

We found little information on mixed (BGI and grey infrastructure) systems, and even less on the optimal combination of BGI and grey infrastructure. Ditthabumrung and Weesakul (2019) proposed a method to quantify the effectiveness of flood management in the Rangsit area in Bangkok, Thailand, including both grey infrastructure (concrete canals) and BGI (retention ponds). Although they quantified the effectiveness of the infrastructure mix, the study does not assess the respective contribution of each type or potential synergies increasing overall effectiveness. In Vietnam, Nguyen et al. (2020) found that expanding pipes was more effective at reducing stormwater volumes than implementing green roofs. In their evaluation of Singapore’s ABC Waters Programme, Lim and Lu’s (2016) examine the combined effect of grey infrastructure (canals and drainage system) and BGI. This follows the Public Utilities Board’s guidelines that only recommends that the overall performance of the system is reported. Combining BGI and grey infrastructure was more common in wastewater treatment systems. For example, domestic wastewater in Can Tho and Ho Chi Minh, Vietnam is passed through a septic tank before being treated in wetland systems (Tran et al. 2019; Zhang et al. 2012). In this case, the differentiation between the impacts of the grey infrastructure (septic tanks) and BGI (wetlands) is easily evaluated separately since water samples were tested for pollutants concentrations before and after each step.

#### Considering Future Climate

The vast majority of studies (94%) did not address climate change or simply mentioned that future climate may exacerbate existing urban water management problems. A notable exception is Wang et al.’s (2016, 2017) simulations of the effectiveness of bioretention in Singapore, which account for different future climate scenarios derived from representative concentration pathways (RCPs). By simulating several shared socio-economic pathways and RCPs for a hypothetical catchment in Singapore, they found that the impacts of urbanization were more ‘adverse than that of climate change’. Kefi et al. (2018) also examined the impacts of climate change on flood risk in Hanoi, Vietnam, and found that total damage from floods may increase by 26% and inundated areas by 19% under future climate. Wolf et al. (2020) also examined the demand for ecosystem-based adaptation based on perceived consequences of natural hazards and climate change. Saltwater intrusion and increased frequency and intensity of floods and droughts were key concerns of local unions and policymakers, leading to high demand for BGI such as mangroves.

#### Assessing Co-benefits

Only a few studies in our review considered co-benefits despite their importance in integrated urban water management. Majidi et al. (2019) assessed the effectiveness of various nature-based solutions based on a range of criteria covering both hydrologic and urban cooling services. Meerow (2019) examined synergistic ‘hotspots’ that maximise co-benefits in Manila and found that the stormwater management service correlated positively with three other services: reducing social vulnerability, reducing the urban heat island effect and improving air quality. Other authors have examined which co-benefits should be prioritised. Balai Kerishnan et al. (2020) surveyed users and non-users of ‘pocket parks’ in Malaysia and found that beyond the hydrological ecosystem services, the most valued co-benefits were stress reduction and provision of a resting space. Alves et al. (2018), with a survey of stakeholders in the Sukhumvit area of Bangkok, Thailand, found that all major stakeholder groups prioritised aesthetics and amenities, but the general public and policymakers placed rainwater harvesting in their top priorities while the scientific community most valued the presence of biodiversity and ecology.

Finally, some authors examined the use of stormwater or wastewater resources for agriculture (Trinh et al. 2013). Most studies and standards caution against the use of stormwater irrigation for gardening (WHO 2017; Payne et al. 2019). Yet they also highlight the potential of this practice with careful crop selection and soil management (Payne et al. 2019, Tom et al. 2014). Ogata et al. (2015) found that rice paddies designed as a wetland filtration system for domestic wastewater could successfully reduce phosphorus and nitrogen levels to meet Thai water standards without compromising the quality and quantity of the rice crops yielded.

#### BGI in Informal Settlements

**Fig. 3:**
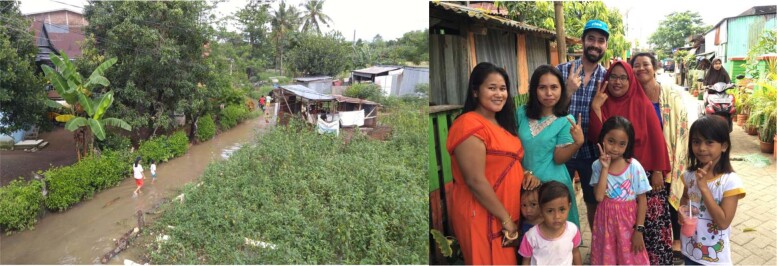
Despite the prevalence of informal settlements in Southeast Asia, there is still a limited understanding of the most appropriate types of natural infrastructure in these environments. Credit: The Revitalising Informal Settlements and their Environments program (RISE; Erich Wolff and Noor Ilhamsyah)

Although there are examples illustrating the potential benefits of rain gardens, riparian vegetation or constructed wetlands around the world (du Toit et al. 2018; Mononimbar 2018), we found very few examples in Southeast Asia. An interesting initiative from the RISE^1^ program aims to demonstrate the application of the water sensitive urban design principles, including the reliance on green infrastructure, to informal settlements in Fiji and Indonesia (Fig. 3). Recent reports from the programme provide examples of engineered systems BGI that are appropriate in informal settlements, and propose a roadmap for leapfrogging (Ramirez-Lovering et al. 2019; Rogers et al. 2019). The researchers recommend mainstreaming of lab testing and field piloting of systems in local conditions. Such recommendations would apply to other examples from practice, such as WetlandsWork^2^ in Cambodia, which confirm an interest in nature-based solutions in the region, without necessarily building the knowledge base due to limited resources for testing and monitoring.

## Discussion

### Scope of the Literature and Limitations of the Review

Not surprisingly, the number of peer-reviewed publications in the region (109) is low compared to the global average: a review by Venkataramanan et al. (2019) found that there were more than 4500 papers on BGI for flood and stormwater management worldwide to be contrasted with 50 papers on BGI in our initial search (Table S2, Supplementary information). Our review suggests that the majority of the scientific knowledge on BGI in Southeast Asia emerges from four of the five wealthier countries in the region (measured by GDP/capita): Singapore, Malaysia, Thailand and Indonesia. This regional disparity mirrors a global trend, suggesting that most of the research on BGI emanates from wealthier countries in the global North (Keeler et al. 2019) and was confirmed by a recent review on urban ecosystem services in Southeast Asia (Lourdes et al. 2021). We found that 40% (21 of 52) of the publications with reported sources of funding had funding from wealthier nations outside the region. This suggests that funders have the potential to reduce the geographic bias by privileging research focusing on low-income countries.

An important finding from the review was that most of the reviewed papers focused on engineered systems (including man-made wetlands) for stormwater and wastewater management, with little information on watershed-scale measures such as forests and rivers upstream of cities. The paradigm shift in urban water management observed globally may explain the amount of research on engineered systems (Brears 2018; Fletcher et al. 2015; Liao et al. 2017; Shuster et al. 2005). The shift promotes a holistic approach to urban water management aiming to mimic the water cycle of a watershed prior to urban development—often relying on engineered systems such as man-made wetlands or bioretention systems (Fletcher et al. 2013; Liu and Jensen 2018). However, our finding points to some blind spots in the integrated watershed management framework. A systemic perspective of the entire watershed, including peri-urban and rural areas, is a critical component of the new urban water management paradigm but this was not apparent in the reviewed studies. Another reason for the bias towards small-scale systems is their lower costs, and hence popularity. Larger river restoration projects, for example (e.g. Bishan-Ang Mo Kio park in Singapore, Dreiseitl et al. 2015), require much larger amount of capital, political will and agency coordination that make them less common.

Although we found some mention of local types of BGI (e.g. ‘monkey cheeks’ in Thailand, biopores and *telajakans* in Indonesia, and rice paddies throughout the region), there is still limited information available on those BGI that are not found in the global North. Lack of knowledge on their role and hydrological performance points to a missed opportunity since these local features are likely to be important in inclusive planning (Frantzeskaki 2019). This is particularly true for rice paddies and urban agriculture, which has potential in Southeast Asia (Agus et al. 2006; Ramirez-Lovering et al. 2019). Given the abundance of rice farming in Southeast Asia—31% of rice harvested globally (Redfern et al. 2012)—the potential of rice paddies both for irrigation water supply and wastewater treatment warrants further investigation. Peri-urban forests also received relatively little attention, which contrasts with the flourishing literature on urban growth and its impacts on ecosystem services (McDonald et al. 2020; Richards et al. 2017).

To contextualize these findings, we note the limitations of the systematic search of the scientific literature. First, our search was limited to English-only literature and references in the Web of Knowledge database. Second, search terms targeted Southeast Asian countries and some studies may not indicate the name of the country or river basin forming part of our search terms (although the indexing system of the Web of Knowledge database minimizes this bias, cf. Supplementary Information). Third, search terms focused on BGI (Search 1, Supplementary Information) and may exclude papers that focused on specialized fields (e.g. stormwater management). Searches with specific types of BGI (e.g. ‘rain gardens’, ‘paddies’) aimed to compensate for this bias and added a significant amount of papers. The selection of search terms is notoriously challenging in the field of BGI given the large amount of overlaps in concepts (e.g., nature-based solutions, green infrastructure, water sensitive urban design, Moosavi et al. 2021; Fletcher et al. 2015). Our search focused on structural elements, although we note that a more deliberate selection of search terms may improve future reviews, especially those with a focus on planning and implementation (see for example the selection process proposed by Adem Esmail and Suleiman 2020). Overall, our systematic search allowed us to retrieve a representative range of papers but does not reflect the full breadth of the scientific production in the region. For example, our review excludes fundamental knowledge relevant to BGI performance, e.g. the emerging amount of research on pharmaceutical pollutant removal in tropical conditions (e.g. Li et al. 2020).

### Hydrologic Performance

Our review confirms the role that BGI can play in urban water management by helping mitigate floods, and treat stormwater and wastewater, in line with the global literature (Fletcher et al. 2015; Liao et al. 2017; Liu and Jensen 2018; Oral et al. 2020; World Bank 2016). For flood mitigation, forest management has the potential to reduce flood risk by reducing peak flows by modest amounts (although up to 39% in the studies reviewed here). This confirms the global evidence that forests reduce flood volume and peak flows (Andreassian 2004; Bradshaw et al. 2007), with the caveat that their effect is limited, or even negative (Sriwongsitanon and Taesombat 2011), for large flood events, when soil saturation capacity is reached. Importantly, even modest reductions in peak flows can have important consequences in highly exposed areas (Lallemant et al. in press). The effect of engineered systems can be more significant, especially for low intensity and frequent flooding in cities, with multiple studies reporting significant amount of reduction from bioretention systems, up to 95% (see Table S3, Supplementary Information). Because these systems are engineered, they can also address large flooding events given enough storage space (e.g. through retention basins).

For water quality, engineered systems including constructed wetlands and bioretention systems show removal rates above 80% for TN and TP (Table S4, Supplemenatry Information), which is above the recommendations from the ABC Waters programme guidelines, themselves derived from Australian guidelines (Public Utilities Board Singapore 2018). However, limited storage capacity leads to lower rates as reviewed by Lim and Lu (2016). Plant selection and design guidelines are now available from recent efforts in Singapore and Australia (Loh 2012; Payne et al. 2019). Almost two thirds (28 of 43) of the evidence for flood reduction comes from modelling studies with limited validation, which questions the evidence base (Brauman 2015). Water quality research was more empirical with the majority (71% of the stormwater quality papers) comprising observed data. For wastewater management, however, empirical data are available for pilots but monitoring of full-scale wetlands remains scarce, reflecting the nascent interest in this type of BGI in the region.

Although information on the hydrological performance of BGI is valuable to water specialists (e.g. stormwater engineers), information on societal and environmental benefits is also needed to mainstream the use of BGI in other professions, e.g. landscape architecture and urban design (Huu Loc et al. 2020). This is particularly true to foster engagement in participatory approaches, as described by some studies in the region (Drosou et al. 2019; Laituri 2020; Møller et al. 2012; Wolf et al. 2020). Yet most scientific papers focus on hydrological functions, overlooking the range of economic or other societal benefits. This blind spot may reflect the infancy of the field—economic valuation studies, in particular, are much more common outside Southeast Asia (US-EPA 2013; World Bank 2017; Gunawardena et al. 2017). It also confirms recent observations in the global literature that there is insufficient consideration of the human health impacts of BGI for stormwater and flood management (Venkataramanan et al. 2019).

### Design and Implementation Factors for BGI

Recent studies have identified insufficient knowledge on implementation or effectiveness as a key challenge to BGI adoption (Sarabi et al. 2020, 2019; Wamsler et al. 2020). In our review of the scientific literature, there was little information on combined grey and BGI systems (and more generally watershed-scale effects), climate change effects, and co-benefits, and informal settlements, with only a dozen studies reported in ‘Design and Implementation Factors for BGI’. We elaborate on these factors in identifying key knowledge gaps in the next section and discuss here why such information is needed for BGI implementation. Studies of combined grey and BGI are important to optimize the implementation of BGI, understanding the location and extent to which BGI can benefit urban water management (e.g. Ditthabumrung and Weesakul 2019; Yi et al. 2020). They can be easily incorporated in ecosystem services assessments to inform urban planning (Adem Esmail and Geneletti 2020; Depietri and McPhearson 2017; Lourdes et al. 2021). Co-benefits of BGI were also poorly considered, while international evidence suggests that they are an important part of the value of BGI and critical to adoption (Frantzeskaki 2019; Keeler et al. 2019). International studies suggest that climate change will reduce the magnitude of hydrologic services (Runting et al. 2017) so regional knowledge should be built to better consider long-term impacts. However, only 6% of the studies incorporated climate change in a quantitative way. Incorporating future climate scenarios in studies is important as the effects of climate change may already be seen in Southeast Asia. For example, an increasing trend in maximum hourly rainfall intensities observed in Singapore (~20% from 1980 to 2012; Yau et al. 2017).

Finally, ecosystem-based management in upgrading informal settlements may contribute to climate adaptation through providing cool spaces and increasing water-related services provided by BGI (Satterthwaite et al. 2020). Yet this opportunity remains a frontier of knowledge and lessons from recent projects are only emerging (e.g. RISE programme, cf. ‘Design and Implementation Factors for BGI’). Evidence outside Southeast Asia suggests that challenges to BGI implementation in informal settlements reflect those in formal areas in cities (Sinharoy et al. 2019). Are highlighted in particular factors related to limited understanding and lack of relevant valuation data on BGI, lack of capacity and expertise, financial barriers, weak governance, lack of baseline data, perception of disservices, limited space competing with other land uses and low climate adaptation capacity (du Toit et al. 2018; Mulligan et al. 2020). Yet scholars note the potential of BGI in so-called leapfrogging strategies, whereby developing countries adopt more advanced water management approaches that avoid issues that developed economies have experienced (e.g. centralized drainage system that reduces water retention services) (Rogers et al. 2019). Further research will help understand the hydrological performance, socio-economic benefits and implementation challenges and opportunities through understanding local governance contexts (Diep et al. 2019; Mulligan et al. 2020; Sinharoy et al. 2019).

### Key Knowledge Gaps

Our discussion highlighted several knowledge gaps and research needs (Table 2). First, there is an urgent need for more empirical data on large-scale effects of BGI to complement and support modelling studies on both flood hazard and water quality issues. This is true for studies examining the effect of the implementation of BGI at the watershed scale, which are notoriously difficult to conduct (Lim and Lu 2016; Walsh et al. 2015), but also for simpler experimental designs comparing watersheds with varying levels of BGI (e.g. comparing land use (Abdulkareem et al. 2018; Asdak et al. 2018)). Such studies would provide evidence to support the debates on the effect of deforestation on flooding, revived for example by the Kalimantan 2021 floods in Indonesia.

**Table 2:** Summary of key knowledge gaps on blue–green infrastructure (BGI) in Southeast Asia

Key knowledge gaps	Recommendations and reference to international research
Building the evidence base:	
What is the large-scale effect (neighbourhood- or city-scale) of implementing BGI for flood risk mitigation and water quality management?	Conduct (at least) short-term monitoring studies of BGI at the watershed or subwatershed scale, to provide evidence of its performance (Walsh et al. 2015)
For engineered systems, what is the long-term performance of BGI for flood risk mitigation and water quality improvement? What are the maintenance requirements?	Conduct long-term monitoring of engineered systems to improve empirical knowledge and validate modelling studies (Hatt et al. 2008)
What is performance of understudied BGI (e.g. rice paddies, forest or river restoration projects) for flood risk reduction and water quality improvement?	Improve understanding of understudied BGI (e.g. rice paddies, peri-urban land use, forest or river restoration projects) or use such as greywater treatment (e.g. Limthongsakul et al. 2017; Payne et al. 2019)
Informing implementation:	
What is the optimal combination of BGI and grey infrastructure in a given neighbourhood or city? In coastal cities, how is stormwater flooding impacted by sea-level rise and what role does BGI play in mitigating flood risk?	Develop modelling capacity and conduct scenario analyses with various combinations of BGI and grey infrastructure (e.g. Joshi et al. 2021)
How will climate change impact BGI hydrologic performance?	Systematically include future climate in modelling scenarios (Zhang et al. 2019)
What are the economic value and co-benefits of main types of BGI in Southeast Asia?	Increase multi-disciplinary collaborations to derive co-benefits and economic values of BGI (Zhang et al. 2020)
What are the most adequate types of BGI in diverse types of informal settlements in Southeast Asia?	Increase collaborations with organisations working with informal settlements to understand challenges and opportunities associated with BGI (Satterthwaite et al. 2020)

Second, some types of BGI are understudied in the scientific literature especially when compared to the breadth of nature-based solutions promoted in the grey literature. Riparian vegetation and river restoration projects are poorly studied in Southeast Asia, despite their potential importance for flood risk mitigation. Rice paddies and urban agriculture, in the form of community gardens or peri-urban individual plots, also require more attention.

Third, engineered systems (e.g. bioretention, green roofs) need to be studied over long term to better understand the decline in performance and maintenance requirements. Issues like clogging, for example, are important considerations for long-term success of engineered systems (Fletcher et al. 2013). More research on the role of greywater is also recommended by some guidance documents, in particular for peri-urban and informal areas. Long-term hydrological impacts should be more routinely translated into societal and environmental impacts to better evaluate their benefits.

Fourth, there is insufficient information for practical design and implementation of BGI. For example, scenarios combining BGI and grey infrastructure or different levels of implementation of BGI would help understand investments needs. Scenarios could also examine other drivers of floods, e.g. subsidence or sea-level rise to understand the potential of BGI under such circumstances. In general, studies should more systematically consider future climate in the experimental design or interpretation of their results, guided by frameworks used by practitioners (e.g. ADB guidelines in Supplementary information). Societal costs and benefits, including additional urban ecosystem services (e.g. heat mitigation) should also be further examined to understand synergies and leverage the multifunctionality of BGI.

Finally, despite the prevalence of informal settlements in the region, a better understanding of the type of BGI recommended in different types of settlements is crucial. Recent initiatives on the potential for leapfrogging of such settlements pave the way for such action research (Rogers et al. 2019). As noted earlier, understanding not only the hydrologic performance but also the local implementation challenges and opportunities is crucial (Diep et al. 2019; Mulligan et al. 2020; Sinharoy et al. 2019). An improved conceptual framing of formal and informal urban water infrastructure (e.g. Adem Esmail and Geneletti 2020) and collaborative work with civil society and NGOs, in particular through upgrading initiatives, will catalyze relevant and legitimate research projects in this field (Satterthwaite et al. 2020).

## Conclusions

In summary, there is evidence of uptake of the concept of BGI for urban water management in research and practice in most countries. With a systematic search focusing on hydrologic performance, we found 109 papers, which should be seen as a lower bar for estimating the total amount of the evidence in the region. These scientific publications also map a network of research institutes, which actively create new knowledge in the region.

Our review confirms that the general principles behind BGI performance apply to Southeast Asian ecological context (climate, vegetation), meaning that there are no technical barriers to using BGI in integrated urban water management in the region. However, there is limited information to design new projects, in part due to a limited amount of empirical data in the region (a majority of studies used models without empirical data or were laboratory studies or prototypes). Factors that need particular attention from a design perspective include climate change, long-term maintenance, combination with grey infrastructure and considerations of BGI that are not studied in the North (e.g. rice paddies) or that are adequate in informal settlements (due to limited space, built infrastructure, or financial resources). We propose directions for research including short- and long-term monitoring programmes, and increasing collaborations with practitioners and ecosystem services scientists, to realize the potential of BGI in the region.

Given the ecological similarities within countries, there are ample opportunities for knowledge transfer within the region. This transfer can take the form of peer-reviewed publications, grey literature (e.g. the ABC Waters programme guidelines or the recent work from the Australia-Indonesia Centre, Payne et al. 2019), conferences and also educational programmes. Similar to international research programmes in the European Union or the United States, coordinated research could significantly increase the body of evidence by leveraging existing efforts and accelerate the mainstreaming of BGI in urban water management in the region.

## Supplementary information

Supplementary Information

Supplementary Tables

## Data Availability

The list of papers reviewed in the article is available in supplementary information.

## Footnotes

1 https://www.rise-program.org/

2 https://wetlandswork.com/products-and-services/sanitation-in-challenging-environments/flood-prone-sanitation-design/)
