# Supplementary material for: Blue–Green Infrastructure for Flood and Water Quality Management in Southeast Asia: Evidence and Knowledge Gaps
Source: Environ Manage. 2021 Apr 16;69(4):699–718. doi: 10.1007/s00267-021-01467-w (PMC9012722; doi:10.1007/s00267-021-01467-w)
Supplement: Supplementary file 1 — Supplementary Information [file 267_2021_1467_MOESM1_ESM.pdf]

Blue-green infrastructure for flood and water quality management in Southeast Asia: evidence and knowledge gaps

Perrine Hamel and Leanne Tan

Supplementary information

1. Country-level statistics for Southeast Asia

Table S1. National statistics for Southeast Asian countries. Sources: https://data.unhabitat.org (% Urban population living in Slums); data.un.org (%urban population, %urban population growth), WHO Joint Monitoring Program: https://washdata.org/data/household (Urban sewerage coverage, except Brunei Darussalam: https://aseaniwrm.water.gov.my/iwrm-in-brunei/, and Malaysia: http://www.jsanic.org/publications/Country_Survey_Reports/Malaysia/JSC_Malaysia_Sanitation_Assessment_Report.pdf); Carrard et al. (2019) (% Urban Population Relying on Groundwater for Domestic Consumption, except Singapore)

Country	Population 2019	% Urban Population 2019	%Urban Population annual Growth[a]	% Urban population living in Slums[b]	% Urban Sewerage Coverage	% Urban Population Relying on Groundwater for Domestic Consumption
Brunei Darussalam	433 000	77.9	1.9	NA	~56	NA
Cambodia	16 486 000	23.8	3.4	45.1	50.8	17
Indonesia	270 626 000	56	2.6	30.6	9.5	90
Lao PDR	7 170 000	35.6	3.2	21.1	1.9	80
Malaysia	31 950 000	76.6	2.7	NA	~70	NA
Myanmar	54 045 000	30.9	1.5	56.1	0	72
Philippines	108 117 000	47.1	2.1	42.9	5.7	NA
Singapore	5 804 000	100	1.7	-	100	0
Thailand	69 626 000	50.7	2.1	23.7	11.9	NA
Timor-Leste	1 293 000	30.9	3.5	33.4	18.2	93
Viet Nam	96 462 000	36.6	3.2	13.8	1.7	46

a. From 2014 to 2019

b. 2018.

2. Scientific Literature search

2.1. Search Overview and Criteria

We conducted a systematic literature review on the Web of Knowledge database from May-June 2020, the final results listed being accurate as of 30 June 2020. We looked for papers reporting on the performance of blue-green infrastructure (BGI), as defined in the Methods of the main text. In each search, we read the titles and abstracts of each paper to determine if they fit the following criteria:

1. BGI must provide water quality management and/or flood mitigation services (excludes coastal flooding and water supply)

2. BGI must be in or directly affecting urban areas within Southeast Asia

3. Papers must either give evidence of BGI hydrologic performance in urban Southeast Asia, design factors or technical considerations for implementation

The selected papers were then read in detail to confirm they met the three criteria. Papers were removed for two main reasons. First, they did not involve urban areas in Southeast Asia. Certain search terms, particularly in wastewater management, tended to return findings about rural villages in Southeast Asia. Most searches returned some studies of countries outside of Southeast Asia that were conducted by or in collaboration with universities from Southeast Asia. Second, papers were removed if they did not include BGI that specifically targeted water management. Some papers covered the benefits of BGI that did not include water management, and others talked about water management using grey infrastructure only. We also looked at the titles of papers in the citations of removed papers, and extracted those that showed potential to be relevant to our aim. They were then read in more detail and selected or removed based on the same criteria.

2.2. Search Terms

Searches were conducted on the Web of Knowledge database, in “All Databases”. The searches were conducted from mid-May to June 2020, with final additions being made on 30 June 2020, through the National University of Singapore Library E-Resources. No advanced filters were used. Specific search terms were tested using trial and error to determine the best searches with enough results to filter through. We conducted the following preliminary search as a wider casting net to determine what types of BGI for urban water management in Southeast Asia were in the literature:

TOPIC: ("flood management" OR "flood mitigation" OR "flood risk") AND TOPIC: ("southeast asia" OR cambodia OR vietnam OR laos OR myanmar OR burma OR thailand OR malaysia OR philippines OR indonesia OR brunei OR singapore OR "east timor" OR "timor-leste" OR mekong OR irrawaddy OR salween OR "chao phraya" OR "red river")

These search terms were searched through titles, keywords, and references titles (https://clarivate.libguides.com/woscc/searchtips). The two authors went through the first 50 results independently and cross-referenced their results with each other to ensure that the same papers and potential search terms were being extracted. The second author went through the remaining 482 results to screen for potential forms of BGI in Southeast Asia that may not have been explicitly referred to with terms more common in literature in the global North, such as BGI or “nature-based solutions”.

The searches in Table S2 below were then conducted based on our findings, with additional terms such as “flood mitigation” or “river” added to filter the results accordingly. Following a revision of the framework, new searches in January and March 2021 were also conducted adding the terms “bioswale”, “retention basin”, “detention pond”, “green roof”, “low impact development”, and “water sensitive urban design” (although only results published before July 2020 were included). These systematic searches yielded a total of 101 papers (Table S2). Eight papers that did not appear in these searches were added to the list, retrieved through previous knowledge or “snowballing”. The final list of papers is available as a Supplementary Table.

Table S2: Summary of Search Terms

All searches include the terms:

TOPIC: ("southeast asia" OR cambodia OR vietnam OR laos OR myanmar OR burma OR thailand OR malaysia OR philippines OR indonesia OR brunei OR singapore OR "east timor" OR "timor-leste" OR mekong OR irrawaddy OR salween OR “chao phraya” OR “red river”)

**No.**	**Search Terms**	**Returns**
1	TOPIC: ("Blue green infrastructure" OR “green infrastructure” OR "natural infrastructure" OR "nature based solutions" OR "river restoration" OR "river rehabilitation" OR bioretention OR “bio-retention” OR biofiltration OR "bio-filtration" OR biopores OR biopore OR "bio pores" OR "bio pore" OR raingardens OR raingarden OR "rain gardens" OR "rain garden" OR “peri-urban forest” OR “peri-urban forests” OR “urban farm” OR “urban farming”)	166 returns,<br>32 relevant papers, 7 retrieved from citations

	Updated searches in January and March 2021 with additional terms (“bioswale” OR “retention basin” OR “detention pond” OR “green roof” OR “low impact development” OR “water sensitive urban design”)	50 additional returns, 11 relevant papers
2	TOPIC: (paddy OR paddies OR wetland OR forest) AND TOPIC: ("flood management" OR "flood mitigation" OR "flood risk")	52 returns, 10 relevant papers
3	TOPIC: (“river” AND “flood”) AND TOPIC: ("river basin management" OR “watershed management” OR “land use management”)	32 returns 5 relevant papers
4	TOPIC: ("wastewater management" OR "wastewater treatment" OR "sewage management" OR "sewage treatment") AND TOPIC: (“wetlands”)	97 returns, 35 relevant papers, 1 retrieved from citations

2.3 Hydrologic performance

For stormwater quantity, quality, and wastewater management, we systematically extracted information on peak flow, runoff retention, and pollutant removal (total nitrogen, phosphorus, and suspended solids—TN, TP, TSS—as well as biological oxygen demand, BOD, for wastewater) from studies reporting empirical data or model results validated for the variable of interest. For example, if the model was validated for water quantity, we do not report results for water quality in this table. Laboratory studies and prototypes were not included in this list of studies. Results for stormwater quantity, quality, and wastewater are provided in Table S3, S4, and S5, respectively. We do not report results for river flood risk reduction given the large variability in contexts (watershed size, amount of forests, rice paddies, etc.), which makes them difficult to interpret.

Table S3: Empirical stormwater quantity performance from peer-reviewed publications

Study	Peak flow reduction	Runoff volume retention
Green roof (Lim and Lu, 2016)	NA	57-68%
Bioretention (Wang et al., 2017)	90-97%	5-95%
Biopores (Setiawan and Rohmat, 2018)	NA	98-100%
Bioretention (Wang et al., 2019)	NA	21% (raingarden)
Green cover (Irvine and Chua, 2016)	NA	[1-75%]
Green roof (Chai et al., 2017)	NA	12-72.5%
Bioretention (Tan et al., 2019)	NA	21-72%

Table S4: Empirical stormwater quality performance (percent pollutant removal) from peer-reviewed publications (TSS: total suspended solids; TN: total nitrogen; TP: total phosphorus)

Study	TSS (%)	TN (%)	TP (%)
Bioretention (Wang et al., 2017)	53	25	46
Bioretention (Muha et al., 2016)	92	[61-65]	[82-83]
Bioretention (Ismail et al., 2014)	81	69	21
Bioretention (Lim and Lu, 2016)	79	NA	NA
Wetlands (Sim et al., 2008)	NA	82	NA
Bioretention (Ong et al., 2012)	[57-73]	[41-64]	[21-53]
Green roof (Rahmah et al., 2015)	NA	NA	[36-89]

Table S5: Empirical wastewater treatment performance (percent pollutant removal) from peer-reviewed publications (TSS: total suspended solids; TKN: total Kjeldahl nitrogen; TP: total phosphorus)

	BOD (%)	TSS (%)	TKN (%)	TP (%)
(Møller et al., 2012)	72	-65	86	15
(Brix et al., 2011)	87	76	38	46

2.4 Funding origins

From the peer-reviewed papers, 51 reported funding sources. Sources varied broadly, with a majority from Malaysia, Singapore, Indonesia, and Thailand, but also European countries, Japan, Korea, the United States. 40% of publications with reported sources of funding were at least partly funded by countries outside the region.

3. Grey Literature Review

To include information published by governments, inter-governmental organisations and other institutions but not in scientific journals, we conducted a grey literature review of BGI for integrated urban water management in Southeast Asia. The searches were conducted using the Google search engine on 3 June 2020 with certain keywords and continued using the snowballing method. The criteria for our grey literature were broader than those for the scientific literature. Criteria 1 and 3 from Supplementary Section 2.1 remained but results did not strictly need to include Southeast Asia. Other results like implementation guides that were relevant to Southeast Asia, such as guides for tropical countries, were also included.

We started our search on websites of relevant inter-governmental organisations and non-governmental organisations, namely the World Bank, Asian Development Bank, World Wildlife Fund, Association of Southeast Asian Nations. The publications on these sites led to work by other organisations, mainly from cited collaborations. We also searched the government

websites and environmental/water agencies of each Southeast Asian country to look for relevant reports. To ensure more coverage, Google searches of:

<Southeast Asian country's name> AND "green infrastructure"" or "<Southeast Asian country's name> AND "nature based solutions"

were conducted, and the first 50 of results for each search were considered. While this method does not aim for exhaustivity, it provides an overview of the available grey literature in the region. It should also be noted that certain websites and documents were only available in non-English languages, and as such could not be included.

This search yielded 27 relevant publications. Documents were split into three categories: implementation guides, city-level strategy reports around urban Southeast Asia, and project-level reports. We found 13 implementation guides that detail factors for planning, implementation and maintenance of BGI for urban water management. 7 of them are global or regional guides, such as the Flood Green Guide by the World Wildlife Fund and the Green City Development Toolkit by the Asian Development Bank. The remaining 6 are guides written specifically by and for certain Southeast Asian cities and/or countries, such as the Active, Beautiful, Clean Water Design Guidelines published by Singapore's Public Utilities Board and Nature-Based Solutions for Cities in Vietnam by the Asian Development Bank. The next category, city-level strategy reports around urban Southeast Asia, consists of 5 reports. These report nature-based solutions strategies in various Southeast Asian countries. Some of them are on BGI for urban water management in particular, such as The Role of Green Infrastructure in Managing Urban Floods [in Can Tho, Vietnam], published by the Institute of Social and Environmental transition. Others about managing urban problems more generally, and feature BGI for urban water management in a particular section, such as Bangkok Resilience Strategy published by 100 Resilient Cities. The last category, project-level reports, includes documents and records that either do not have the primary purpose of explaining BGI for urban water management in Southeast Asia, or records that are less formal. The 9 documents include a range of formats including evaluation documents, press releases and blog posts by credible organisations.

References

Brix, H., Koottatep, T., Fryd, O., Laugesen, C.H., 2011. The flower and the butterfly constructed wetland system at Koh Phi Phi-System design and lessons learned during implementation and operation. Ecol. Eng. 37, 729–735. https://doi.org/10.1016/j.ecoleng.2010.06.035

Carrard, N., Foster, T., Willetts, J., 2019. Groundwater as a source of drinking water in southeast Asia and the Pacific: A multi-country review of current reliance and resource concerns. Water (Switzerland) 11, 1605. https://doi.org/10.3390/w11081605

Chai, C.T., Putuhena, F.J., Selaman, O.S., 2017. A modelling study of the event-based retention performance of green roof under the hot-humid tropical climate in Kuching. Water Sci. Technol. 76, 2988–2999. https://doi.org/10.2166/wst.2017.472

Irvine, K.N., Chua, L.H.C., 2016. Modeling Stormwater Runoff from an Urban Park, Singapore Using PCSWWM. J. Water Manag. Model. 25. https://doi.org/doi.org/10.14796/JWMM.C410

Ismail, A.F., Sapari, N., Wahab, M.M.A., 2014. Vegetative swale for treatment of stormwater runoff from construction site. Pertanika J. Sci. Technol.

Lim, H.S., Lu, X.X., 2016. Sustainable urban stormwater management in the tropics: An evaluation of Singapore's ABC Waters Program. J. Hydrol. https://doi.org/10.1016/j.jhydrol.2016.04.063

Møller, K.A., Fryd, O., De Neergaard, A., Magid, J., 2012. Economic, environmental and socio-cultural sustainability of three constructed wetlands in Thailand. Environ. Urban. 24, 305–323. https://doi.org/10.1177/0956247811434259

Muha, N.E., Sidek, L.M., Jajarmizadeh, M., 2016. Water Quality Improvement through Reductions of Pollutant Loads on Small Scale of Bioretention System, in: IOP Conference Series: Earth and Environmental Science.

Ong, G.S., Kalyanaraman, G., Wong, K.L., Wong, T.H.F., 2012. Monitoring Singapore's first bioretention system: Rain garden at Balam estate. WSUD 2012 - 7th Int. Conf. Water Sensitive Urban Des. Build. Water Sensitive Community, Final Progr. Abstr. B.

Rahmah, A.K., AB Ghani, A., Zakaria, N.A., Shaharudin, S., 2015. Efficiency of intensive green roof in high intensity rainfall for stormwater treatment: selection of vegetations, in: 36th IAHR Congress.

Setiawan, I., Rohmat, D., 2018. Test the Effectiveness of Biopore in the Framework of Eco-Campus Development at Universitas Pendidikan Indonesia. IOP Conf. Ser. Earth Environ. Sci.

Sim, C.H., Yusoff, M.K., Shutes, B., Ho, S.C., Mansor, M., 2008. Nutrient removal in a pilot and full scale constructed wetland, Putrajaya city, Malaysia. J. Environ. Manage. 88, 307–317. https://doi.org/10.1016/j.jenvman.2007.03.011

Tan, K.M., Seow, W.K., Wang, C.L., Kew, H.J., Parasuraman, S.B., 2019. Evaluation of performance of Active, Beautiful and Clean (ABC) on stormwater runoff management using MIKE URBAN: a case study in a residential estate in Singapore. Urban Water J. 16, 156–162. https://doi.org/10.1080/1573062X.2019.1634744

Wang, J., Chua, L.H.C., Shanahan, P., 2019. Hydrological modeling and field validation of a bioretention basin. J. Environ. Manage. 240, 149–159. https://doi.org/10.1016/j.jenvman.2019.03.090

Wang, J., Chua, L.H.C., Shanahan, P., 2017. Evaluation of pollutant removal efficiency of a bioretention basin and implications for stormwater management in tropical cities. Environ. Sci. Water Res. Technol. 3, 78–91. https://doi.org/10.1039/c6ew00285d
